# Left behind on the path to 90‐90‐90: understanding and responding to HIV among displaced people

**DOI:** 10.1002/jia2.26031

**Published:** 2022-11-09

**Authors:** Tetyana I. Vasylyeva, Danielle's Horyniak, Ietza Bojorquez, Minh Duc Pham

**Affiliations:** ^1^ Division of Infectious Diseases and Global Public Health University of California San Diego San Diego California USA; ^2^ Public Health Discipline Burnet Institute Melbourne Victoria Australia; ^3^ School of Public Health and Preventive Medicine Monash University Melbourne Victoria Australia; ^4^ Department of Population Studies El Colegio de la Frontera Norte Tijuana Mexico

**Keywords:** displaced people, forced migration, HIV care continuum, HIV prevention, HIV treatment, lower‐ and middle‐income countries

## Abstract

**Introduction:**

In 2021, the number of people affected by displacement worldwide reached the highest on record, with an estimated 30.5 million refugees and 4.6 million asylum seekers seeking safety across international borders and further 53.2 million people displaced within their countries of origin. Most forcibly displaced persons come from or relocate to lower‐ and middle‐income countries (LMICs) and many of those countries have large HIV epidemics. In this commentary, we describe some of the challenges at the intersection of HIV and displacement vulnerabilities that cannot be easily addressed in resource‐limited environments.

**Discussion:**

HIV transmission and prevention and treatment efforts in the context of displacement are affected by myriad behavioural, social and structural factors across different stages of the displacement journey. For example, structural barriers faced by people experiencing displacement in relation to HIV prevention and care include funding constraints and legal framework deficiencies. Such barriers prevent all forced migrants, and particularly those whose sexual identities or practices are stigmatized against, access to prevention and care equal to local residents. Xenophobia, racism and other social factors, as well as individual risky behaviours facilitated by experiences of forced migration, also affect the progress towards 90‐90‐90 targets in displaced populations. Current evidence suggests increased HIV vulnerability in the period before displacement due to the effect of displacement drivers on medical supplies and infrastructure. During and after displacement, substantial barriers to HIV testing exist, though following resettlement in stable displacement context, HIV incidence and viral suppression are reported to be similar to those of local populations.

**Conclusions:**

Experiences of often‐marginalized displaced populations are diverse and depend on the context of displacement, countries of origin and resettlement, and the nature of the crises that forced these populations to move. To address current gaps in responses to HIV in displacement contexts, research in LMIC, particularly in less stable resettlement settings, needs to be scaled up. Furthermore, displaced populations need to be specifically addressed in national AIDS strategies and HIV surveillance systems. Finally, innovative technologies, such as point‐of‐care viral load and CD4 testing, need to be developed and introduced in settings facing displacement.

## 1 INTRODUCTION

Over the past decade, conflict, persecution and disasters have driven over 100 million people from their homes [1, 2]. In 2021, the number of people forcibly displaced, those forced to leave their homes as a result of armed conflict, situations of generalized violence, human rights violations or natural or human‐made disasters [3], reached 84 million worldwide, the highest on record, with 36 million people seeking safety across international borders, and 48 million internally displaced people (IDP) displaced within their countries of origin [4]. In 2022, this number was further inflated by the 10 million people displaced by the war in Ukraine [4]. Although the far‐reaching social and economic impacts of displacement have been well documented, it is only in recent years that the health impacts of forced migration have been recognized as a global priority, with urgent calls to build the evidence base for understanding its health‐related drivers and outcomes [5].

In 2021, 85% of displacement events occurred in low‐ and middle‐income countries (LMICs), and the majority of displaced persons were hosted in LMICs neighbouring their countries of origin [4]. Several of these countries also had large populations of people living with HIV (PLWH) and/or HIV responses that were not on track to reach 90‐90‐90 targets. The direction and scale to which displacement affects HIV risk in displaced populations depends primarily on HIV epidemic characteristics in both host and origin locations, on the context and stage of displacement. For example, in settings where displacement is caused by an armed conflict, HIV vulnerability differs at three stages of the displacement journey, defined as pre‐flight, transit and post‐flight, corresponding to periods before displacement, during displacement and after resettlement. As such, HIV incidence might increase pre‐flight compared with times preceding the conflict [6], but evidence shows that HIV prevalence does not increase during periods of conflict [6, 7, 8] and there might in fact be an inverse relationship between HIV incidence and conflict intensity [6]. Disrupted healthcare, interactions between military and civilians, and sexual violence can increase HIV risk at all stages of the displacement journey during a conflict [9, 10].

In this commentary, we provide an overview of structural, social and individual factors that affect HIV transmission and progression along the HIV care continuum for forcibly displaced people. We then explore opportunities for comprehensive and sustainable responses to improve health outcomes for affected populations, such as the inclusion of displaced people in national AIDS strategies and the implementation of innovative research approaches, such as point‐of‐care (POC) testing.

## 2 DISCUSSION

### 2.1 HIV vulnerability in the context of displacement

**Figure 1:**
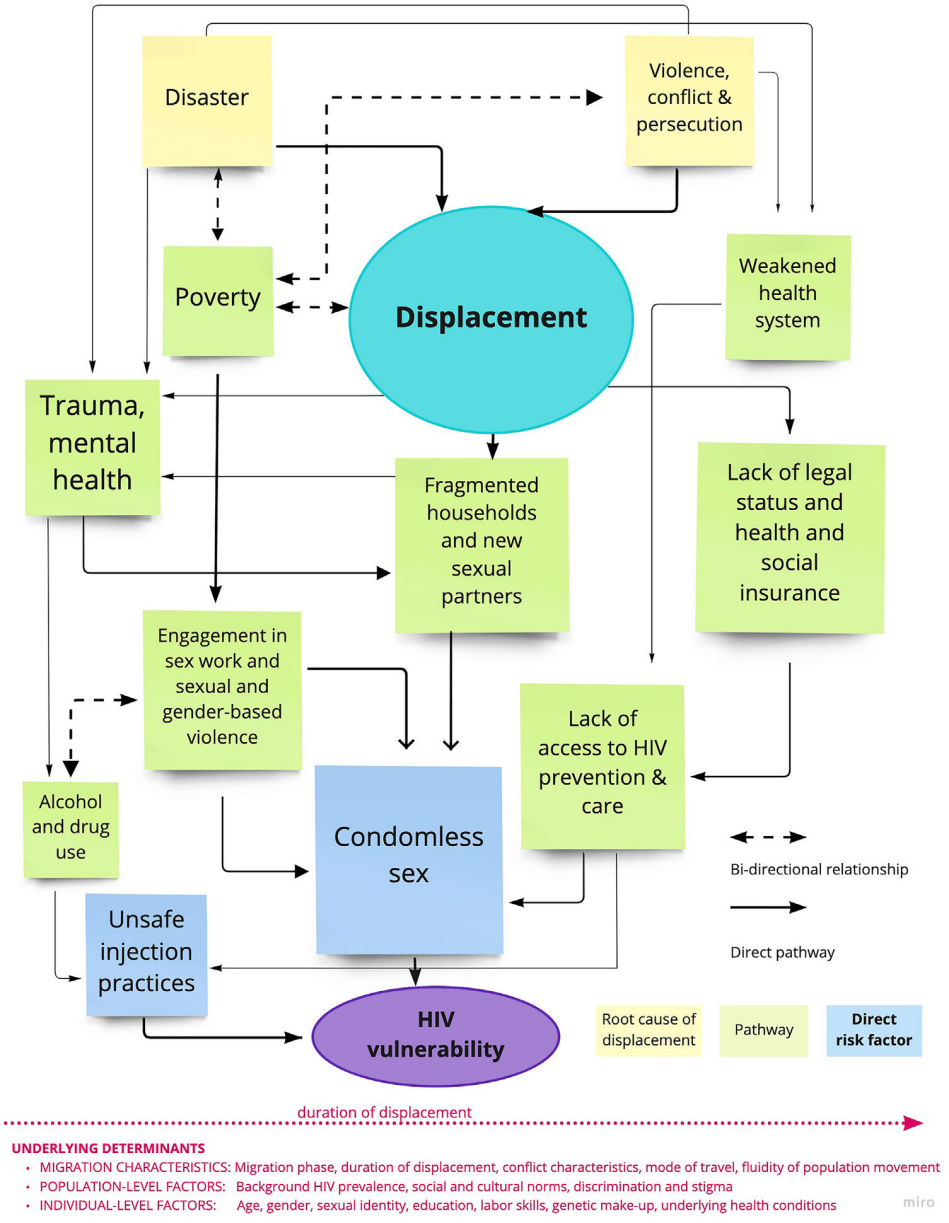
Relationships between displacement and HIV vulnerability. Here, we define “Root cause of displacement” as a set of factors directly preceding displacement that caused displaced people to move; “Pathway” is defined as a set of factors that can increase vulnerability to HIV at any stage of the displacement journey; and “Direct risk factor” as behaviours that directly enable HIV transmission.

Appeals for increased attention to forced migration and health have called for the integration of social determinants of health approach [11, 12]. Building from work examining the social determinants of migrant health [13, 14] and reviews of HIV among various groups of migrants [15, 16, 17, 18], we provide here an overview of the structural, social and behavioural factors which shape HIV vulnerability for displaced persons (Figure 1).

#### 2.1.1 Structural factors

Structural factors, particularly those operating at the “macro” level (i.e. socio‐political and economic context), create HIV risk environments through their impacts on social processes and individual risk and protective behaviours at all stages of the displacement journey [19]. For example, funding constraints have been identified as a factor impacting the delivery of HIV services for crisis‐affected populations [20, 21]. With most displaced persons coming from and hosted in LMICs where resources may be limited, further burdens are placed on already underequipped systems. In Lebanon, Syrian refugees have access to services provided by the National AIDS Control Program, but the health system is already overstretched and expensive, leading some refugees to rely on care illegally provided by Syrian healthcare workers, and contributing to a growing HIV epidemic among men who have sex with men [22, 23].

Exclusionary legal and policy contexts create structural barriers to health by limiting access to social security, health insurance and healthcare. Only 10, primarily high‐income, countries globally adopt a comprehensive approach that provides forced migrants with rights equal to citizens (although several others provide equality on paper but not in practice); in many settings, refugees only have access to emergency care or have conditional access to care (i.e. only allowed to use services in assigned locations) [24]. Even in contexts where legal access to health services is provided, service utilization may be constrained by other structural barriers, such as the complexity of refugee status determination processes [25], and inadequate integration policies which fail to address social and economic disadvantage, leading displaced people to prioritize needs, such as housing and employment over health [26]. Low health literacy, language barriers and lack of culturally appropriate services have also been identified as key structural barriers to service utilization [13]. Furthermore, criminalization of people on the basis of their sexual identity, gender expression or engagement in sexual practices—policies that are still in place in more than 60 countries [27]—perpetrates a climate of hate, discrimination and persecution, and produces vulnerability to HIV through an interplay with social factors that can obstruct access to HIV prevention and care services at all stages of displacement [28, 29].

#### 2.1.2 Social factors

Recent years have seen many countries grappling with xenophobia and anti‐immigrant sentiment [30, 31]; although attitudes towards refugees are generally more positive compared with other migrants, concerns about deservingness, crime, security, integration and economic burden persist [30, 31, 32], despite evidence both from macro‐economics and modelling that long‐term refugees have a positive impact on local economies [33, 34]. Xenophobia and racism have both been identified as determinants of health [35, 36], contributing to experiences of stigma and discrimination. This is particularly salient in the context of infectious diseases, with displaced people commonly framed as dangerous and diseased, a threat to host populations and a burden on health systems [37]. There is considerable overlap between beliefs underlying stigma relating to forced migrant status and stigma relating to HIV status or engagement in HIV risk behaviours [38, 39], with these dual stigmas working together to create barriers to health service access and utilization, and ultimately contribute to poor health outcomes among forced migrants [29, 40, 41].

Sexual violence and exploitation, a frequent companion of military conflicts and an independent HIV risk factor, is disproportionally targeted at women, girls and persons with diverse sexual and gender orientations and expressions [28, 42, 43]. Although data are limited and sexual violence often goes unreported [20], studies show that up to one‐fifth of female refugees, IDP and asylum seekers have experienced sexual violence, including rape [43, 44]. Vulnerability continues through all three stages of the displacement journey: at least half of all sexual violence events happen during transit [45] and sexual exploitation continues in refugee‐hosting contexts post‐flight [42]. Conditions of distress and displacement in transit and post‐flight settings may create threats to masculinity which drive acts of sexual violence as an effort to reclaim power and control [46].

#### 2.1.3 Individual and behavioural factors

The structural and social factors outlined above may create direct risk factors for HIV or precursors to an engagement in HIV risk behaviours. For example, following displacement journeys, households become fragmented and family and social relationships disrupted, potentially reshaping norms around sexual behaviour and leading to the establishment of new sexual partnerships [47]. Although studies have documented associations between mobility, sexual risk behaviours, such as sex with casual partners and multiple concurrent partnerships, and HIV acquisition, findings in the context of displacement are inconclusive [15, 48]. Some studies have reported high engagement in sexual risk behaviours among refugees [49], while others found no significant difference in the prevalence of multiple partnerships and casual sex between refugees and community residents [8]. Concerningly, a recent study among Senegalese refugees in Mauritania noted a belief that HIV‐positive refugees were prioritized for resettlement, leading some to purposefully engage in behaviours that increase the odds of acquiring HIV [29]. There is considerably more evidence, however, that displaced persons may be exposed to HIV through engagement in sex work or the informal exchange of money, employment, housing, goods or services for sex [42, 43, 50, 51], particularly in situations where risk may be further exacerbated in the context of substance use or when everyday survival needs shape decision‐making with respect to condomless sex [51].

Poor psychosocial health and mental wellbeing arising from the traumatic events many displaced people have experienced [52, 53] and exacerbated by discrimination, barriers to social and economic inclusion, and difficulties accessing healthcare services [5, 13] may be associated with HIV risk behaviours, particularly sexual risky behaviours, such as less frequent condom use, among forced migrants [18, 54]. Alcohol and drug use can be a form of coping with traumatic experiences and other stressors in contexts of displacement [55], which can in turn increase the probability of engaging in behaviours that increase HIV risk [53].

### 2.2 The HIV care continuum in the context of displacement

#### 2.2.1 HIV diagnosis

In transit and post‐flight, even in settings where displaced populations are eligible to access healthcare, barriers to testing and diagnosis exist [56] and include a lack of information about where to obtain testing, prioritization of basic needs over health, financial and logistical constraints, difficulty navigating complex administrative processes, language and communication challenges, stigma, concerns about the confidentiality of health information and mistreatment by healthcare providers [29, 41, 57, 58, 59]. On the other hand, when HIV testing is mandatory as part of the resettlement pathway, those with precarious legal status may fear being denied a visa or deported if diagnosed with HIV [60, 61]. Populations in transit and those in situations of ongoing instability may also be impacted by attacks on healthcare workers and health facilities which hinder service provision and deter healthcare seeking [62].

There is a dearth of intervention research addressing the HIV care continuum among displaced populations, but some interventions that showed to be effective in increasing HIV testing and diagnosis include outreach interventions, clinic‐based routine testing programmes in refugee settlements, home‐based testing programmes, self‐testing and mHealth approaches [57, 63, 64, 65, 66].

#### 2.2.2 Antiretroviral therapy (ART) initiation and adherence

On a structural level, high adherence and good clinical outcomes in displaced people might be attributed to strong regional collaboration and contingency planning, such as providing patients with surplus medication in case of disruptions [67]. In Ukraine, where a recent escalation of the war has forced more than 10 million people to flee their homes and is threatening to significantly affect the HIV epidemic in the country, public health organizations have been coordinating with medical suppliers, AIDS clinics and non‐governmental organizations to assess the remaining stock of medications in order to keep an uninterrupted provision of HIV care and opioid agonist therapy, though provision challenges remain in this fast‐evolving situation [68, 69]. Facilitating treatment adherence in dynamic situations, where periods of renewed or heightened conflict may be unpredictable, presents a significant challenge, particularly with regard to drug‐resistant mutations arising as a result of treatment interruptions [70]. Among displaced populations in stable displacement contexts and those resettled in high‐income countries, viral suppression rates on par with host nationals are achieved through equitable access to HIV support and care [56, 71, 72], offering evidence to underpin calls to scale up treatment in this group [73]. Research on displaced people in Malaysia and Kenya showed that intervention to increase individual's resilience and reduce food and health insecurities might help improve ART adherence [74]. Furthermore, both people with a history of displacement and PLWH are more likely to have symptoms of depression and anxiety [52, 53], which can be another barrier to treatment adherence [40, 75].

### 2.3 Harnessing momentum to achieve HIV equity

Recent years have seen a proliferation of high‐level commitments to addressing forced migration and health, providing a strong platform for national and global action [76, 77, 78]. To help progress these efforts, we suggest the following priority areas for action.

First, a stronger evidence base is required to guide HIV responses. Significant knowledge gaps persist, both in relation to HIV epidemiology and progress along the HIV care continuum. To facilitate the monitoring of HIV epidemiology trends, efforts must be made to include displaced populations in national planning as currently few national AIDS strategies explicitly address IDP or refugees [79]. Furthermore, national HIV monitoring and surveillance systems must be adapted to better include displaced populations [80]. Targeted research, in particular studies to measure progress along the care cascade, identify barriers and facilitators associated with the care cascade and trial interventions to address them, is also essential. While such studies have been conducted in stable displacement contexts [71, 72, 74] and in high‐income countries [81], there is still a critical need for similar research in LMICs and countries experiencing acute conflict.

Innovative research approaches which can be utilized to generate nuanced insights and inform targeted prevention strategies include phylogenetic and phylodynamic analysis of HIV sequences to study phylogenetic clustering in migrant communities, providing insights into when and where transmissions occurred, but so far, these efforts were mostly limited to labour migrants [82, 83]. Rapid, portable and relatively cheap genetic sequencing technologies (i.e. MinION from Nanopore) have also shown promise in viral infections monitoring in resource‐limited settings [84] and could be used in field settings to generate insights into the dynamics of chronic viral infections transmission in forced migrants [85], although their application for HIV is still in development.

New technologies to improve the delivery of prevention, treatment and care responses in displacement settings, particularly in LMICs, include HIV self‐testing (HIVST), a process in which a person collects their own sample (oral fluid or whole blood), performs the test and obtains the result independently at a place and time of their choice. HIVST can be employed as an approach to close the HIV testing gap among priority populations, including displaced people [86]. Compared with standard HIV testing, HIVST can increase the uptake and frequency of testing by 45‐200% [87].

Finally, POC technologies have been widely used to support the scale‐up and decentralization of HIV treatment and care in LMICs and may have similar potential for displacement settings, such as refugee camps, as they eliminate the need for highly trained healthcare professionals and laboratory facilities. POC CD4 testing can be employed to inform clinical decision‐making prior to ART initiation. Currently available POC CD4 tests perform well compared with standard laboratory‐based cytometry in field conditions [88], are feasible to be integrated into routine clinical care [89] or can be easily performed by lay healthcare workers, and can improve patients’ linkage to care and clinical outcomes [90]. POC technologies also have the potential to expand viral load monitoring coverage, enhance the efficacy of HIV treatment and improve retention in care and viral suppression by eliminating the need for expensive instruments which can only be used in centralized laboratories [63, 91].

## 3 CONCLUSIONS

Despite the pledge for equitable access, displaced populations are being neglected in efforts to end the AIDS epidemic. Forced migrants experience unique and complex circumstances which place them at risk of HIV and continue to be adversely impacted by preventable barriers to accessing prevention, treatment and care, particularly at the structural level. Building on the momentum gained in recent years, we must move beyond all‐encompassing approaches addressing the needs of forced migrant populations and ensure equitable inclusion and facilitate targeted policy, planning and responses.

## COMPETING INTERESTS

There are no competing interests.

## AUTHORS’ CONTRIBUTIONS

DH wrote the first draft of the manuscript. TIV prepared the final draft of the manuscript. IB and MDP contributed to writing sections of the manuscript. All authors have read and approved the final manuscript.

## FUNDING

Dr. Vasylyeva is supported by the Branco Weiss Society in Science Fellowship. Dr. Horyniak is supported by an Australian National Health and Medical Research Council Early Career Fellowship (grant 1092077).
